# From Friends across the Sea

**Published:** 1887-11-05

**Authors:** 


### FROM FRIENDS ACROSS THE SEA.

BY OUR READERS' RELATIVES AND COLONIAL COUSINS.

**All can help.**

In another column, page 105 of this number, will be found a proposal to encourage families who keep up an active correspondence with their friends in other lands to make a selection from the letters they receive for publication in THE HOSPITAL. With a view of showing the kind of letter which is desired we publish the following communication from a clergyman's son in Australia. It is desirable that the friends should give the writer a distinct name, and it would add to the interest if the first communication was prefaced by a short biographical account of the writer on the lines here given.

**Robert's Early Adventures in Australia.**

Robert was the second of three sons, his father, a clergyman, had died before he left school, and his mother's means were too limited to remain in England and farm as he desired. It was therefore decided that he could have his wish if he would go to Australia, where he was to be taught all the business of a squatter by a gentleman whom his friends heard of through an advertisement in the *Times.* A premium was paid, and the gentleman in question stated he had come over to England to conduct Robert and two other young fellows to his run in Victoria. On the voyage out their conductor did his best to induce them to play cards with him, and so to obtain possession of such money as they might have. Fortunately, our young colonist saw through the scheme and refused to play, but he did not gain much by his foresight, as he was induced by his conductor to deposit all his money with him for safe keeping (?), and he never saw it again. Arrived in the colony, every opportunity was afforded the young Englishmen for dissipation and indulgence. After remaining at one of the first hotels in —— for three weeks, their conductor disappeared and left them to their fate. Robert then made an effort to find work of some kind, and on his return from one of these expeditions he found that his companions had also disappeared, having first taken his letters of introduction from his portmanteau. Thus Robert was left without money or introductions in a strange land, and to his horror the landlord presented him with the bill for the whole period of their residence, no portion of it having been paid. The landlord was fortunately sympathetic, and under the circumstances behaved fairly enough by giving Robert a small sum of money and retaining most of his luggage. Thus a lad of seventeen years of age found himself landed in Australia, and compelled to turn his hand to anything he could find to do. He communicated at once with his friends in England, but long before they could send him any assistance he was reduced to doing odd jobs and even to stone-breaking. The discomforts and disadvantages incidental to such an occupation can be imagined. Driven almost to despair, he one day received a request to call at an address given in answer to one of the numerous letters he had sent in reply to advertisements in the papers. The request came from a member of the Colonial Legislature, and the interview resulted in Robert being taken on as a shepherd. He soon found himself at the squatter's station, and at work upon a run, but his position was not that his friends paid a large sum to obtain for him. The member of the Legislature stated to the young settler's brother years afterwards that the reason he decided to take the lad was the reply Robert gave when asked to stop and take lunch with his family. "No, sir; my present position and rough workman's dress do not fit me to meet your daughters at the present time, but I am very hungry and shall be glad to have some food in the kitchen." From that time the young Englishman devoted himself to a squatter's life. He soon rose to be the manager of the station, and ultimately married the niece of his original employer.

**Freddy Joins Robert.**

Freddy, the writer of the following letter, is the brother of the man whose early experiences are here described. Freddy joined his brother years afterwards, and has become thoroughly attached to the life. He is twenty-two years of age, and his daring and spirit may be realised from the following anecdote. After residing at a station for many months he rode one day into the nearest large town, and found there was to be a ball there in three days' time. The ladies, being in despair for want of flowers, came to Freddy for advice, and he undertook to provide them in time for the ball. It afterwards appeared he had seen a number of flowers up country on his way down. He therefore rode back more than a day's journey, secured the flowers, and brought them to the ladies late on the afternoon of the ball, having been in the saddle for thirty-six out of the forty-eight hours during which he was absent. Freddy found, however, that despite his deserved popularity, he could not keep his eyes open for long together, and so at nine o'clock he had to retire to bed. A year ago, at Freddy's earnest request, his friends sent him some money to enable him to procure a run to work for himself. Unfortunately, the money arrived while he was in one of the capital towns of Australia, where some horsedealers induced him to purchase a horse depository with a portion of it, and so he lost half his capital in a few months. This so disgusted him that he set off with his twenty horses for the gold fields in Western Australia, the following letter being written by him from Lady Carrington's Reef, Cambridge Gulf.

**A Prosaic Reef Owner.**

"When I come to look back I feel awfully ashamed of myself for not writing before. I could have written two letters since my last, but not being able to send you good news, thought it better to wait. Even now I am afraid my news is not of the best; there are so many difficulties to contend against, yet I have settled down into a prosaic reef owner, and after the adventurous and stirring life I have led for the last twelve months it is decidedly monotonous. I hold a sixth share in the gold reef, whose name is on the heading of this letter. We shall make some hundreds of pounds apiece out of her, no matter how things turn out, and so far the chances are we may make as many thousands. The stone we have at grass now, some thirty tons, is all very payable stone, and when a machine arrives we shall get a good return for our labour, but I am afraid we shall have a hard struggle to keep going till then. Up till quite lately I have been out exploring and prospecting new country for gold, an adventurous and stirring life that seems made for me, notwithstanding the hardships you have to put up with.

**Three Weeks on Skilly.** Once we lived for three weeks and some days on skilly, a delightful compound of flour and water. The recipe for making it is extremely simple. Try and see how it goes. To two pinches of flour add one pint of boiling water. Filling, but not strengthening. I can strongly advise a course of it for liver complaint. Our camp is pitched at the foot of a valley dividing the ranges of mountains, from which some of the largest rivers in the Kimberley country take their rise. Close to us is a large stagnant creek, which is not running. Hence the nearest pure water is two miles distant, but the want of it does not trouble us much, as we have to send the horses down to water every day, and the black boy fills bags of canvas with water, each holding five gallons, which he packs back on his return.

**Australian Scenery.** This is not a particularly picturesque place, though there are one or two lovely spots in the neighbourhood. Notably a gap in the Albert Edward Range, some five miles distant, through which there are some splendid springs of water running. On entering it the walls of the range rise up precipitously on each side for some hundreds of feet, while before you are dozens of small cascades tumbling down in all directions out of caves in the solid rocks from under great boulders, while scattered about everywhere are hundreds of cabbage tree palms of all sizes, from the piccaninny of ten feet high to the old warriors of a hundred and a hundred and fifty feet in height. If you happen to stop and examine any of the deep pools formed here and there, you will see the festive crocodile taking his siesta, always with one eye open however, on the off chance that some unwary duck might alight near where he is basking. They are harmless as regards ourselves, but I hate the sight of the brutes for all that. They remind me too much of the alligator, but we are too far inland for those huge pests of tropical rivers.

**Native Hecatombs.** I must not forget to mention that the caves at the Palm Springs, the place I have just tried to describe and most signally failed to do, are, or were, used as hecatombs by the Myal blacks. In nearly every one of them you will find numerous bundles of bones, carefully wrapped up with a species of bark called paper bark, and which has the appearance of matting. Unless you knew what they were you could take no objection to them, as they are so neatly bound up with paper bark and tied with rope made from the hair of the dead, that you might take them for brown paper parcels stowed away in the caves for security. It seems strange in the nineteenth century to be surrounded by "Myalls," Aboriginal for wild blacks. They are all cannibals, and as regards physical form, perfection, but their faces are anything but perfect.

**Lynch Law.** One of my mates was speared by them and two others wounded when we first found gold here. There were only twenty-four men on the field then, so it was a serious matter. We formed ourselves into two parties. One tracked the murderers down, and shot them next day; the other took a tour round, and shifted every nigger they could see within ten miles of the camp. They are a cowardly, skulking lot, and unless they can sneak on you and catch you unawares they will not interfere with you. I have a good dog and a good repeating Winchester rifle, which carries thirteen cartridges, besides a Colt's revolver; so with three such trusty friends there is not much to fear if you are anything like prepared. As the tame black boy is waiting with the horses to take my note, I must here conclude this letter."

